# The North American Medico-Chirurgical Review

**Published:** 1857-02

**Authors:** 


*The North American Medico-Chirurgical Review* is the title of a new bi-monthly, published in Philadelphia, by Lippincott, and edited by our friends Profs. S. D. Gross and T. G. Richardson. It is a combination of the former Louisville Review and the Philadelphia Medical Examiner, and makes a splendid typographical appearance, being a portly octavo of 160 pages.

Before welcoming the new friend to our exchange list, we must express our regret at the loss of Dr. Hollingsworth from the fraternity of editors. A fine scholar, a practical common sense man, and a finished writer, he is regarded with respect and affection by all who know him. His retirement is in accordance with a determination long since expressed.

Of the "Review" we can only say that the character and experience of its editors are such as to encourage the hope that it will be the best journal in the country. The first number—unlike most first issues—is full of merit, and we predict for it a brilliant career. Let us go farther. We have frequent letters from subscribers asking us what *other* journal they shall take. We now say invest four dollars in the "Review," for we have a sincere wish that the hiatus it proposes to fill should be occupied.

